# Psychological and social challenges of patients with locally advanced and metastatic gastrointestinal stromal tumours (GIST) on long-term treatment with tyrosine kinase inhibitors: a qualitative study with patients and medical oncologists

**DOI:** 10.1007/s00520-023-07810-7

**Published:** 2023-05-26

**Authors:** Deborah van de Wal, Lena Fauske, Øyvind S. Bruland, Robin L. Jones, Bernd Kasper, Roger Wilson, Winette T. A. van der Graaf, Olga Husson

**Affiliations:** 1 Department of Medical Oncology, The Netherlands Cancer Institute-Antoni van Leeuwenhoek, Amsterdam, The Netherlands; 2 Department of Oncology, Norwegian Radium Hospital, Oslo University Hospital, Oslo, Norway; 3 Department of Interdisciplinary Health Sciences, Institute of Health and Society, University of Oslo, Oslo, Norway; 4 Institute of Clinical Medicine, University of Oslo, Oslo, Norway; 5 Sarcoma Unit, The Royal Marsden NHS Foundation Trust, London, UK; 6 Division of Clinical Studies, The Institute of Cancer Research, London, UK; 7 Sarcoma Unit, Mannheim University Medical Center, University of Heidelberg, Mannheim, Germany; 8 NCRI Consumer Forum, Sarcoma Patients Euronet, Shropshire, UK; 9 Department of Medical Oncology, Erasmus MC Cancer Institute, Erasmus University Medical Center, Rotterdam, The Netherlands; 10 Department of Psychosocial Research and Epidemiology, The Netherlands Cancer Institute, Amsterdam, The Netherlands; 11 Department of Surgical Oncology, Erasmus MC Cancer Institute, Erasmus University Medical Center, Rotterdam, The Netherlands

**Keywords:** Gastrointestinal stromal tumours, Tyrosine kinase Inhibitors, Psychological issues, Social life, Psychosocial issues, Qualitative research, Interviews

## Abstract

**Purpose:**

Tyrosine kinase inhibitors (TKIs) have revolutionized the treatment of locally advanced and metastatic gastrointestinal stromal tumours (GISTs). Patients are experiencing prolonged survival but often at the expense of their health-related quality of life. It is not only the physical side effects that impact GIST patients’ daily lives but also the psychological and social challenges they have to deal with. This qualitative study aimed to explore the psychological and social life challenges of GIST patients with locally advanced and metastatic disease on ≥ 5 years TKI treatment.

**Methods:**

Semi-structured interviews with 15 locally advanced and/or metastatic GIST patients and 10 medical oncologists with experience of delivering care to this specific patient group were conducted. Thematic analysis was used to interpret the data.

**Results:**

Psychological challenges expressed by participants concerned fears, scanxiety, negative change in emotion and mood, doubts about their treatment and follow-up, living with uncertainty, lack of understanding from others or healthcare professionals, and constantly being reminded of their illness. Challenges regarding social health included financial difficulties, challenges in relationships, concerns about fertility and parenting, work, and impact on social activities.

**Conclusion:**

The reported psychological and social challenges can significantly hamper the overall quality of life of GIST patients. Some challenges were clearly underreported and hardly recognized by medical oncologist, as they may tend to focus on the physical side effects and clinical outcomes of treatment. Therefore, it is essential to take the patient’s perspective into account in research and clinical practice to ensure optimal care for this patient group.

**Supplementary Information:**

The online version contains supplementary material available at 10.1007/s00520-023-07810-7.

## Introduction

Gastrointestinal stromal tumour (GIST) is a rare cancer, with an incidence of 10–20 people per million per year in Europe [1, 2]. GIST can arise anywhere in the gastrointestinal tract, and the tumour can remain asymptomatic for a long time. For that reason, it is not uncommon for GIST to have already metastasized to the peritoneum or liver at the time of diagnosis [2]. The introduction of tyrosine kinase inhibitors (TKIs) in 2001, specifically imatinib, dramatically changed the prognosis of patients with locally advanced and metastatic GIST as the median survival improved from approximately 12 to 57 months [3]. Today, 10–15% of imatinib-treated patients with metastatic GIST are still responding to imatinib after 10 years of treatment [4]. In the event of disease progression or intolerance to imatinib, sunitinib, regorafenib, and ripretinib are registered as second-, third-, and fourth-line therapies [5].

As a result of the extended survival, for locally advanced and metastatic GIST, the term ‘chronic’ cancer has been introduced, even though the vast majority of patients are incurable, even on chronic TKI treatment [6, 7]. Most patients with locally advanced or metastatic GIST will eventually succumb to their disease, and the fear of disease progression is undeniably a challenge for most of these patients [8]. In general, the widespread impact of receiving a GIST diagnosis and its treatment on health-related quality of life (HRQoL) are largely overlooked. HRQoL is a multidimensional concept that includes the patient’s perception of the impact of the disease and its treatment on physical, psychological, and social functioning [9]. Most studies in GIST patients focused on clinical outcomes (e.g. response rate, time to progression, progression-free survival) and medical side effects of treatment, only a few studies also addressed HRQoL in a more holistic way [10].

Qualitative research among GIST patients is even more scarce. A recent study found that more than half of the GIST patients with metastatic disease experienced side effects that influenced their daily lives in negative and challenging ways, which urged them to adapt to ‘a new normal’ [11]. In another qualitative study, GIST patients expressed how tiredness, impaired memory, and physical challenges were among the detrimental impacts of the disease on their family life, vocational life, social life, and leisure time [12]. Both studies underline that patients with metastatic GIST struggle with the side effects of TKI treatment and consequences of living with chronic cancer. In other words, patients are experiencing prolonged survival but often at the expense to HRQoL.

To create a complete overview of the impact of the disease and its treatment on HRQoL, it is essential to take the patient’s perspective into account. Patients not only experience physical side effects, but also face psychological and social challenges that influence their daily lives, something that is currently understudied. In a previous qualitative study we focused on the physical challenges of patients, describing the patient’s perspective on side effects of TKIs in the treatment of locally advanced and metastatic GISTs [13]. Here, we will focus on the psychological and social challenges of GIST patients with locally advanced and metastatic disease during TKI treatment.

## Methods

### Participants

Patients with a locally advanced or metastatic GIST treated with a TKI for ≥ 5 years were invited for an interview. Patients were recruited from the Netherlands Cancer Institute/Antoni van Leeuwenhoek Amsterdam, the Erasmus MC Rotterdam, and Radboud UMC Nijmegen, all in The Netherlands. Medical oncologists from different countries, including the Netherlands, United Kingdom, Germany, and Norway, with experience of delivering care to this specific patient group were interviewed. Recruitment continued until data saturation, the point during the interviews where no additional issues were being found, was reached.

### Procedure

The study was approved by the local Institutional Review Board (IRBd20-083). All participants were given verbal and written information about the study, and written informed consent was obtained from all patients that participated.

### Interviews

All interviews were conducted by the first author (D.v.d.W.) following a semi-structured interview schedule. The interviews were either conducted face-to-face at the hospital or at the home of the patient, via Teams or over the telephone. Patients were asked to consider their experiences from the moment they first got symptoms, when they received the diagnosis, started treatment, up to the present day. To address all domains of the biopsychological model, patients were specifically asked about their physical, mental, and social challenges. Prior to the interview, patients completed a short questionnaire about sociodemographic, tumour, and treatment characteristics. Medical oncologists were asked to describe the issues they encountered during the follow up visits with GIST patients. Both the patient and medical oncologist interview schedules are available as supplementary material 1.

### Data analysis

With permission, interviews were recorded and transcribed verbatim. Transcripts were independently reviewed by two reviewers (D.v.d.W. and M.J.P.R.) using Nvivo software (version 12) [14]. The transcripts were analysed according to the principles of thematic analysis [15]. To become familiar with the data the reviewers read the transcripts repeatedly. Sections that were related to the study objectives were highlighted and coded into the three main themes of the biopsychological model: physical, psychological, and social health. Within these main themes, the reviewers independently defined subthemes. In the interviews patients also acknowledged positive issues as a result of their GIST diagnosis and treatment. However, we decided to exclude these positive issues as they are not part of the construct of HRQoL [16]. Afterwards, the reviewers discussed their findings, refined the subthemes, and resolved differences until consensus was reached. By using this inductive way of analysis, separate HRQoL themes were identified for GIST patients on long-term treatment. All quotes were anonymised. To ensure rigour in our study the ‘Consolidation Criteria for Reporting Qualitative Studies’ (COREQ) guidelines were followed [17], the checklist is available as supplementary material 2.

## Results

### Participants

Twenty-five interviews were conducted, 15 with GIST patients and 10 with medical oncologists. Of the interviewed patients, 53% were males, the mean age at diagnosis was 48.1 years (range 13–64 years), and the time since diagnosis was 13.2 years. Patients were treated with TKIs for locally advanced (*n* = 1) or metastatic (*n* = 14) GIST for a mean duration of 11 years (range 5–20 years). All patients had been given imatinib as a first-line treatment, and 3 patients had progressed onto second-line sunitinib. The majority of patients (*n* = 13) reported experiencing side effects of their TKI treatment. All sociodemographic, tumour and treatment characteristics are presented in Table 1. The interviewed medical oncologist all had 10 to 30 years of experience in delivering care to GIST patients.

**Table 1:** Socio-demographic, tumour and treatment characteristics of interviewed GIST patients

	Patients (*n* = 15)
Sex, *n* (%)	
Male	8 (53%)
Female	7 (47%)
Age at interview in years, mean ± SD	61.3 ± 12.2
Age at diagnosis in years, mean ± SD	48.1 ± 13.7
Marital stage, *n* (%)	
Married/living with partner	10 (67%)
Divorced/separated	1 (7%)
Widowed	2 (13%)
Single	2 (13%)
Highest education, *n* (%)	
Secondary school	1 (7%)
Secondary (vocational) education	9 (60%)
Higher Vocational Education and university	5 (33%)
Comorbidity, *n* (%)	
None	2 (13%)
1	7 (47%)
2	0 (0%)
3	2 (13%)
≥ 4	4 (27%)
Time since diagnosis in years, mean ± SD	13.2 ± 4.7
Location primary GIST, *n* (%)	
Stomach	8 (53%)
Small intestine	7 (47%)
Other	1 (adnexa)*
Surgery for primary GIST, *n* (%)	
Yes	14 (93%)
No	1 (7%)
Time since previous surgery in years, mean ± SD	12.8 ± 5.9
Diagnosed with metastasis, *n* (%)	
Yes	14 (93%)
No	1 (7%)
Time since diagnosis of GIST metastasis in years, mean ± SD	11.3 ± 5.0
Current stage, *n* (%)	
Locally advanced GIST	1 (7%)
Metastatic GIST	14 (93%)
Current type of treatment, *n* (%)	
First-line imatinib	12 (80%)
Second-line sunitinib	3 (20%)
Time on treatment in years, mean ± SD	11.0 ± 4.6
Time on imatinib in years, mean ± SD	10.0 ± 5.1
Time on sunitinib in years, mean ± SD	5.0 ± 4.6
Side effects of treatment, *n* (%)	
Yes	13 (87%)
No	2 (13%)

*One patient had a primary GIST located in the stomach and an extra abdominal location in the adnexa at time of diagnosis

### Interviews

Interviews were conducted between March 2021 and September 2021. The average duration of the patient interviews was 70 minutes, ranging from 24 to 120 min. The duration of the interviews with medical oncologists ranged from 20 to 34 min with an average of 27 min.

### Psychological health

**Fig. 1:**
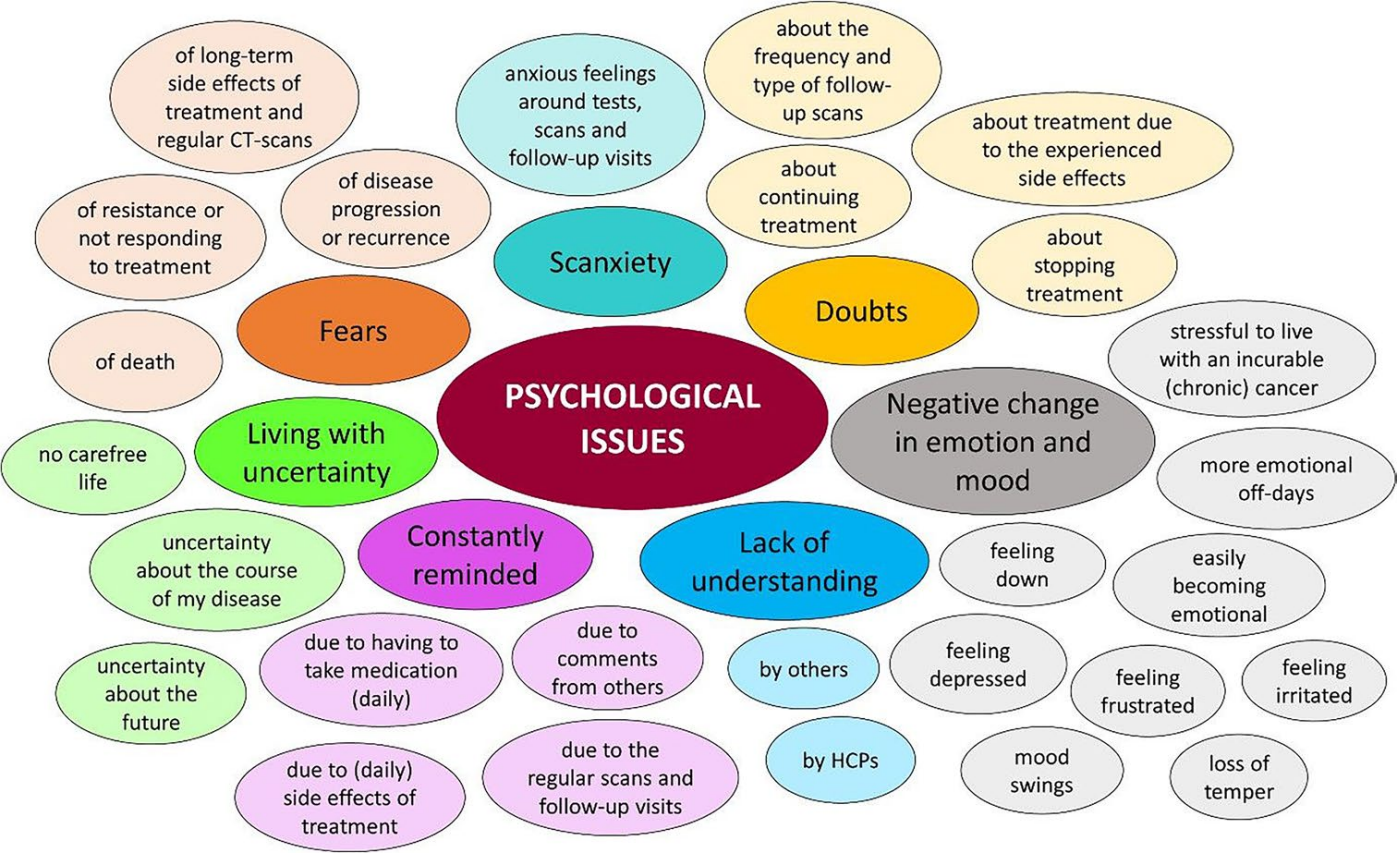
Psychological issues experienced by GIST patients—darker coloured circles present the subthemes, codes expressed by participants within these subthemes are shown in the lighter coloured circles

Within the main theme psychological health, we defined seven subthemes: fears, scanxiety, constantly reminded, doubts, negative change in emotion and mood, lack of understanding, and living with uncertainty. ‘Scanxiety’ is a relatively new term and defines anxious feelings and distress around regular tests, scans and follow-up visits [18]. An overview of examples of psychological issues expressed by the participants within the subthemes is presented in Fig. 1, and a frequency table is available as supplementary materiel 3.

The subthemes along with codes and quotes are presented in Table 2. The numbers in parentheses denote the in-text reference for the quote in the table. Both patients (*n* = 12) and medical oncologists (*n* = 8) expressed fears about the disease becoming resistant to TKI treatment (1.1.1 and 1.1.2), disease progression (1.2.1–1.2.3), death (1.3.1 and 1.3.2) and disease activity when experiencing a physical sensation (1.4.1 and 1.4.2). Interestingly, patients (*n* = 5) experienced fear of side effects on the long run of their TKI treatment and regular CT scans (1.5.1) with contrast fluid, an issue which medical oncologists did not report. In addition, the majority of patients emphasised feeling anxious around regular tests, scans and follow-up visits (2.1.1 and 2.1.2). Scanxiety was also described and recognized by almost all medical oncologists (2.1.3).

**Table 2:** Quotes of participants

Subtheme	Code	In-text reference	Quotes
Fears	Fear of resistance or not responding to treatment	1.1.1	“The only fear I have, is that it [the imatinib] will lose its effect. And then what happens? So as long as it works, I’m happy, but I know there’s a chance that’s going to happen. At least that’s what they [the oncologists] always tell you.” Patient 13—71-year-old female on imatinib for 9 years
		1.1.2	“Maybe they will develop resistance and things like that, that can induce fear.”—Medical oncologist 9
	Fear of disease progression or recurrence	1.2.1	“Then I turned out to have a low imatinib blood level. So then the oncologist said ‘we should increase the dose, but we don’t really know if that makes sense.’ In the end I chose to do that, because I do have fear of progression: ‘suppose I didn’t do it and I activated the tumour cells again’, yes, then I wouldn’t be able to forgive myself.” Patient 1—59-year-old female on imatinib for 17 years
		1.2.2	“Of course, in the beginning, people are also very worried about fear of recurrence.”—Medical oncologist 8
		1.2.3	“The sword of Damocles, it’s going well now, but how long will it go well?”—Medical oncologist 7
	Fear of death	1.3.1	“My biggest fear the moment I was diagnosed was, ‘Damn, I might only have a few more years to live and I have kids and I’m a single parent.’” Patient 2–57-year-old male on imatinib for 9 years
		1.3.2	“Yes, of course that varies a lot, the fear of ‘will I survive this?’—Medical oncologist 5
	Fear of disease activity when experiencing a physical sensation	1.4.1	“...that you feel something strange in your body, which I had with my shoulder, and every time of course I think that it is the tumour. And then it turns out to be nothing.” Patient 12—54-year-old male on imatinib for 12 years
		1.4.2	“With every little thing they feel: is it [the disease] back or does it have something to do with it? We know this very well from all kinds of patients, of course.”—Medical oncologist 5
	Fear of long-term side effects or consequences of treatment	1.5.1	“Mainly I think: what does it [the treatment] do in the long run? Because I notice that my complaints are now just getting more intense, I think: is that my age or is it the Glivec that has been working on me for so long that the complaints I already had are getting nastier? Especially the decrease in muscle strength.” Patient 11 – 68-year old female on imatinib for 19 years
Scanxiety	Feeling anxious around tests, scans and follow-up visits	2.1.1	“I’ve had dozens of scans and yet every time I think, by the look of the radiologist, the way I am being called in to the doctor’s office...this is not good” Patient 12–54-year-old male on imatinib for 12 years
		2.1.2	“Especially the time between the scan and the result. Well, that’s agony.” Patient 10—45-year-old female currently on sunitinib after 10 years of imatinib
		2.1.3	“I think, a lot of patients have that sort of constant fear of the scans being bad.”—Medical oncologist 10
Constantly reminded	Due to (daily) side effects of treatment	3.1.1	“With Glivec, it was my appearance, the swollen eyes and the eye bleeds. And people asking about it, yes.” Patient 10—45-year old female currently on sunitinib after 10 years of imatinib
	Due to comments from others	3.2.1	“Some patients suffer from eye edema, which is particularly disfiguring and leads to comments, which patients experience as annoying.”—Medical oncologist 2
	Due to having to take (daily) medication	3.3.1	“People who are reminded of being a cancer patient every time because of their daily medication intake.”—Medical oncologist 4
	Due to the regular scans and follow-up visits	3.4.1	“Having to go to the hospital and always undergoing CT scans, and then every time being confronted with the fact that they are really a patient while they don’t feel like a patient at all.”—Medical oncologist 7
Doubts	Doubts about stopping treatment	4.1.1	“I’m afraid it [the tumour] would grow again if I stopped. I’d rather keep taking them [the pills], then I’m sure that tumour will stay away.” Patient 5—67-year-old female on imatinib for 8 years
		4.1.2	“Yes, you also know that if it [the disease] comes back then you have more metastases at that time. And, what you also know from that French study… that Imatinib is slightly less effective in people who restart treatment.” Patient 2—57-year-old male on imatinib for 9 years
		4.1.3	“Actually, most people just want to continue treatment because that gives them security. Just stopping is what makes people feel uncomfortable.”—Medical oncologist 6
		4.1.4	“I happened to be talking about that with my treating physician, to stop the medication, to see what happens. … I actually take my pills very irregularly and nothing ever changes, so I once said to him: ‘ If only we could just stop and see what happens’ ” Patient 14—53-year-old male on sunitinib for 9 years
	Doubts about continuing treatment	4.2.1	“Of course it is the case, that if you take that medication for a long time, then at a certain point you do not really know what would happen if you stopped taking it. And yes, I often wonder: is treatment still needed?” Patient 1—59-year-old female on imatinib for 17 years
	Doubts about treatment due to the experienced side effects	4.3.1	“I don’t know if it’s just me, but some drugs are worse than the disease, aren’t they? What I take is just pure poison for your body.” Patient 14—53-year-old male on sunitinib for 9 years
	Doubts about the frequency and type of follow-up scans	4.4.1	“They always did follow-up with an MRI scan and at one point they found a questionable lesion. Then they did a PET scan and it turned out that there was a recurrence behind my stomach.” Patient 3—30-year-old male on sunitinib for 6 years after 11 years of imatinib
Change in emotions and mood	Feeling down	5.1.1	“It hit me terribly because I was always a sportsman, I did a lot of sports and at a certain point I couldn’t anymore. And it made me really grumpy and down too.” Patient 7—73-year-old male on imatinib for 9 years
	Feeling depressed	5.2.1	“When I look at the quality of life, then at some point you just have to bite the bullet and accept that your life has changed forever. And so that’s something, I don’t know what it was, but that’s was a very difficult period. I’ve been very depressed.” Patient 11—68-year-old female on imatinib for 19 years
		5.2.2	“I have had a few people who have developed serious depressive symptoms. Sometimes to the point where you have to interrupt the medication. If only because they have the feeling that it also has something to do with it, or that they just can’t handle the fatigue or nausea at such a moment. That it is just too much together.”—Medical oncologist 5
		5.2.3	“Patients really had psychological problems. Depression is something which we see under imatinib. And we even had a suicide once. It is something, which is not very well documented and described in the literature. But there are cases.”—Medical oncologist 3
	Easily becoming emotional	5.3.1	“I have become more emotional. I tear up, because of anything. I can’t stand conflicts either. Those are things that are the result of, I think, taking the drug.” Patient 8—71-year-old male on imatinib for 5 years
	Loss of temper	5.4.1	“Our eldest granddaughter sometimes acts up a bit... He sometimes finds it difficult to deal with that, and then loses his temper.” Patient 8—71-year-old male on imatinib for 5 years
	Mood swings	5.5.1	“I sometimes suffer from mood swings. Sometimes I can get really upset. When I’m driving a car or something, then I can suddenly go completely out of my mind.” Patient 15—69-year-old male on imatinib for 5 years
	Feeling frustrated	5.6.1	“The side effect of fatigue unfortunately is really there every day. It frustrates you, to think: I’m 68, I sometimes function as a female of 75 or 80 years old. And I fight against it, but it always remains.” Patient 11—68-year-old female on imatinib for 19 years
	Having more emotional off-days	5.7.1	“I’m now also in a phase of my life that I can say, ‘I’m having an off-day, I will stay in and do nothing’. Better tomorrow.” Patient 11—68-year-old female on imatinib for 19 years
	It is stressful to live with an incurable chronic cancer	5.8.1	“It is of course a psychological rollercoaster for these people, the group of long responders. First you think ‘I will die’, then you think ‘I will live’, then you actually know that you will die but you don’t know when.“—Medical oncologist 7
Lack of understanding	By others	6.1.1	“So with everything, he thought that my concerns were actually unjustified, so to speak.” Patient 12—54-year-old male on imatinib for 12 years
		6.1.2	“You don’t look ill, even if you have a tumour that a lot of people know about.” Patient 6—73-year-old female on imatinib for 18 years
		6.1.3	“The expectation was that someone with a large or metastatic tumour would die within a short period of time, and then he or she does well on imatinib for a long time, which sometimes causes misunderstanding.”—Medical oncologist 4
	By healthcare professionals	6.2.1	“Then he said ‘oh, then I’ll just declare you 100 percent incapacitated for work again, because in six months you won’t be there anymore.’ And I thought that was a very blunt statement at the time.” Patient 1—59-year-old female on imatinib for 17 years
Living with uncertainty	No carefree life	7.1.1	“You have lost your worry-free attitude a bit, but if you start worrying about that, you have no life anymore.” Patient 8—71-year-old male on imatinib for 5 years
	Uncertainty about the future	7.2.1	“I can’t make plans for the future. But in terms of a few months, that is still possible.” Patient 15—69-year-old male on imatinib for 5 years
	Uncertainty about the course of my disease	7.3.1	“It still troubles me that I was given a life expectancy of four years. And am I now in my extra remaining-remaining-remaining time or something?” Patient 3—30-year-old male on sunitinib for 6 years after 11 years of imatinib
		7.3.2	“And the turmoil of not knowing how long it [the treatment] will work.”—Medical oncologist 5
		7.3.3	“That starts when you take it [the imatinib] for about ten years, then you suddenly become conscious; ‘There is no one in a similar situation’.” Patient 11—68-year-old female on imatinib for 19 years
Impact on social activities	Difficulty going out in public because of having to deal with my disease or the side effects	8.1.1	“Well, I am less active socially. The fatigue takes away your motivation. So I skip things more often, then I think I’ll just taking my rest, and nothing to worry about.” Patient 12—54-year-old male on imatinib for 12 years
		8.1.2	“They are terrified that they will start playing bridge and get diarrhea.”—Medical oncologist 1
	Had to change or give up my hobbies	8.2.1	“I don’t go swimming anymore, and I think that’s a pity, actually. But I also don’t want to get sick every time, I don’t feel like that either.” Patient 6—73-year-old female on imatinib for 18 years
		8.2.2	“I don’t do that anymore, no voluntary work for example, because it was no longer possible. I miss it, because I really enjoy connecting with people.” Patient 11—68-year-old female on imatinib for 19 years
	Had to negatively adjust social activities	8.3.1	“Yes, almost always. You sometimes have younger people or very active people who, for example, do a lot of sports. Yes, that really does decrease.”—Medical oncologist 5 and 6
		8.3.2	“So I’ll go, but I won’t go as long as I’d like. I would prefer to stay put until the very end, but I don’t anymore.” Patient 15—69-year-old male on imatinib for 5 years
		8.3.3	“Yeah, maybe at a slightly slower pace, otherwise I can’t keep up. But well, that could also depend on my age.” Patient 6—73-year-old female on imatinib for 18 years
	Always having to plan activities and/or planned activities are always subject to change	8.4.1	“...then I’ve made an appointment, then I have to cancel it because then I just can’t do it. It is always conditional. We like to do things, go out together and stuff. But we’ve already figured it out, if we’ve planned several things in a row then I’m just really tired on the second or third day.” Patient 7—73-year-old male on imatinib for 9 years
	Unable to function in big groups	8.5.1	“I am also less good in big groups. That’s one thing. I used to be able to do that easily, but now I don’t feel so comfortable there. No, I feel closed off.” Patient 4—71-year-old female on imatinib for 9 years
Relationships	Relationship difficulties with your partner	9.1.1	“Relationship problems that have arisen because the dynamics in their relationship have changed. For example, before they were always the stronger one in the relationship and now the weaker as a result of the GIST and the treatment.”—Medical oncologist 2
	Not able to find a partner	9.2.1	“Well, a fifty-year-old man likes movies, going out, travelling, has metastatic cancer and a good sense of humour. No it doesn’t work.” Patient 2—57-year-old male on imatinib for 9 years
	Lost friends	9.3.1	“Well, in the beginning when I was very sick, I lost a lot of them. But those might not be real friends either.” Patient 4—71-year-old female on imatinib for 9 years
	Feeling a burden to others	9.4.1	“I felt very guilty towards other people. Almost like I caused panic and they must be thinking: that panic-monger, he’s still there, what was he talking about?” Patient 12—54-year-old male on imatinib for 12 years
		9.4.2	“Now I know my friends think; if he cancels, he has a good reason. In the beginning I thought, if I was too tired to come, people would say: ooh, there you have him again. And that was a nagging thought.” Patient 7—73-year-old male on imatinib for 9 years
Fertility and parenting	Worried about becoming infertile and not being able to have children	10.1.1	“And the fertility issues around it, that’s really something. Most people with a GIST are older, so then that’s not an issue, but I have a few younger patients where it is a really big issue.”—Medical oncologist 7
		10.1.2	“Having children is something that I think is problematic in my current situation.” Patient 3—30-year-old male on sunitinib for 6 years after 11 years of imatinib
	Difficulty starting a family	10.2.1	“Firstly, regarding IVF, that [door] was already closed by then, because I didn’t want to continue the injections endlessly. Then we were really far along in the adoption process when 2011 came around…so I got that tumour again, so well, that was also over.” Patient 10—45-year old female currently on sunitinib after 10 years of imatinib
Financial difficulties	Less income	11.1.1	“But that‘s definitely an issue. I mean, they lose their jobs. They get a reduced pay, or whatever. And so, they of course, have less money than they had before. And that definitely is an issue for a lot of them.”—Medical oncologist 3
		11.1.2	“I do have financial difficulties due to the Imatinib. As I said, my career has ended and now I am selling puzzles. Well that‘s definitely not enough to live on.” Patient 2—57-year old male on imatinib for 9 years
	Not able to buy a house	11.2.1	“Often enough we have considered buying another house, but yes, I did feel hindered in that.” Patient 1—59-year old female on imatinib for 17 years
		11.2.2	“You are marked as palliative, so yes, you will no longer get your mortgage insurance.” Patient 3—30-year-old male on sunitinib for 6 years after 11 years of imatinib
	More expenses on health insurance	11.3.1	“The deductible for the chronically ill is a real shame. I understand that the deductible should be a discouragement for the excessive use of care, but for people with a chronic condition I think this is an extra penalty.” Patient 2—57-year-old male on imatinib for 9 years
Work	Not able to work full-time	12.1.1	“I now work five hours a day. In fact, they said I didn’t have to work anymore. They wanted to declare me incapacitated for work, but I said, I don’t want that. As long as I can still do it, I’ll just keep working." Patient 14—53-year-old male on sunitinib for 9 years
	Change jobs	12.2.1	“So I was a postdoc and what I’m not going to do anymore is stand in a lab six days a week and work to the bone. I made a very conscious choice, that was partly due to the circumstances, but I now do administrative work.” Patient 12—54-year-old male on imatinib for 12 years
	Lost jobs	12.3.1	“At some point I found out that I was going to be fired, but they hadn’t discussed it with me at all.” Patient 6—73-year-old female on imatinib for 18 years
	Feeling as if you have no career opportunities	12.4.1	“When I look at my career, it’s gone because of the GIST and the imatinib, that remains extremely hard.” Patient 2—57-year-old male on imatinib for 9 years

Some patients (*n* = 4) acknowledged constantly being reminded of their illness due to the daily side effects of treatment (3.1.1), comments from others (3.2.1), having to take medication (daily) (3.3.1) or regular scans and follow-up visits (3.4.1).

Patients (*n* = 7) expressed multiple doubts while on treatment, most frequently about stopping their TKI. They underlined the fear of progression when stopping (4.1.1), and the fear of not responding to treatment when needing treatment again (4.1.2). However, none of the patients actually had stopped taking TKIs because they felt more secure while being on treatment (4.1.3). Doubts about discontinuing treatment due to long-term absence of disease activity were regularly discussed with medical oncologists in clinical practice (4.1.4). Patients wondered if the treatment was still effective or needed (4.2.1). Three patients also doubted treatment because of the side effects they experienced as they expressed that the side effects were maybe worse than the disease itself (4.3.1). Two patients described their doubts about the frequency and type of follow-up scans carried out (4.4.1), driven by the possibility that progression would not be detected in time.

A negative change in emotion and mood was reported by 10 patients and 8 medical oncologists. GIST patients most frequently reported feeling down (5.1.1), feeling depressed (5.2.1) including suicidal thoughts, and easily becoming emotional (5.3.1). Some patients experienced loss of temper (5.4.1), mood swings (5.5.1), frustrations (5.6.1) and more emotional off-days (5.7.1) while being on treatment. Merely feeling down and depressed (5.2.2 and 5.2.3) were recognised by oncologist. Furthermore, only medical oncologists mentioned living with an incurable chronic cancer as being stressful (5.8.1).

Seven patients experienced a lack of understanding, either by others (e.g. by doing well or better than expected or not looking ill) (6.1.1–6.1.3) or by healthcare professionals (HCPs) (6.2.1).

Patients described living with uncertainties including not living a carefree life (7.1.1), uncertainty about their future (7.2.1) and uncertainty about the course of their disease. Regarding the uncertainties about the course of their disease, patients expressed not knowing what will happen now they passed their prognosis (i.e. given life expectancy at time of diagnosis) (7.3.1), not knowing how long treatment will be effective (7.3.2) and not knowing any ‘examples’ referring to fellow GIST patients in the same situation they are in (7.3.3).

### Social health

**Fig. 2:**
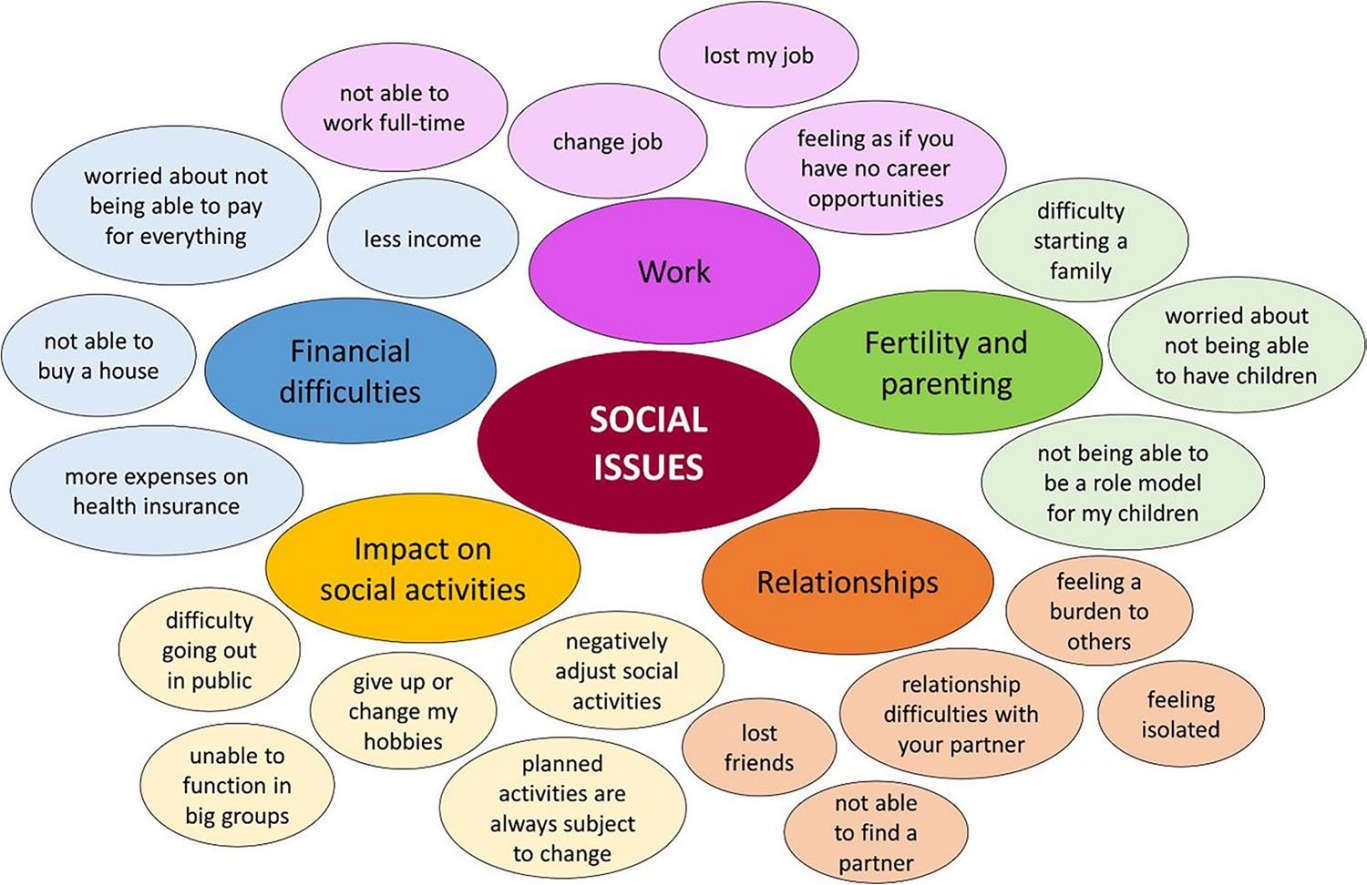
Social issues experienced by GIST patients—darker coloured circles present the subthemes, codes expressed by participants within these subthemes are shown in the lighter coloured circles

In the main theme social health, we defined five subthemes: impact on social activities, relationships, concerns about fertility and parenting, financial difficulties and work. An overview of examples of social issues expressed by the participants is presented in Fig. 2, and a frequency table is available as supplementary materiel 4.

Both patients (*n* = 10) and medical oncologists (*n* = 9) acknowledged the negative impact of being a GIST patient on TKI treatment on social activities. Medical oncologists reported that patients experience difficulty going out in public because of having to deal with their disease or the side effects (8.1.1). According to medical oncologists, the most troublesome side effects that prevented patients from going out were diarrhoea (8.1.2) and the constant fatigue, both also mentioned as troublesome by patients. Patients underlined that they had to change or give up their hobbies (8.2.1 and 8.2.2) or had to negatively adjust their social activities (8.3.1), including shorten the duration (8.3.2) or slowing down during activities (8.3.3). In addition, three patients expressed having to plan activities or that planned activities are always subjected to change due to the experienced, sometimes unexpected, side effects (8.4.1). Two other patients had trouble functioning in big groups, and described not feeling comfortable (8.5.1).

Issues regarding relationships were mentioned frequently by both patients (*n* = 10) and medical oncologists (*n* = 9). Medical oncologists mostly described relationship difficulties with partners (9.1.1), while patients expressed not being able to find a partner (9.2.1) or losing friends (9.3.1). Patients also emphasised feeling a burden to others for various reasons, among which, still being alive while diagnosed with cancer (9.4.1) or having to cancel activities with friends due to side effects such as fatigue (9.4.2). Some participants, 3 patients and 1 medical oncologist, expressed concerns about fertility and parenting. They expressed worries about becoming infertile and not being able to have children (10.1.1 and 10.1.2), difficulty starting a family (10.2.1) and not being able to be a role model for their children.

Financial difficulties were also frequently mentioned (6 patients, 3 medical oncologists), often as a result of less income (11.1.1 and 11.1.2) which coincided with concerns about being able to pay for everything. A reduced income was usually work-related, patients expressed not being able to work full-time (12.1.1), having to change jobs (12.2.1) or losing their job (12.3.1). Patients (*n* = 2) also felt they did not have career opportunities (12.4.1), especially compared to their ‘healthy colleagues’. Two patients also underlined the inability to buy a house (11.2.1) due to difficulties with insurance and mortgages as a result of being a palliative cancer patient (11.2.2). Some patients also acknowledged the higher expenses on health insurance because of being chronically ill (11.3.2).

## Discussion

This qualitative study explored the psychological and social life challenges of GIST patients with locally advanced and/or metastatic disease on long-term TKI treatment, both from the perspective of the patients, as well as medical oncologists with experience of delivering care to this specific patient group. While being on treatment GIST patients can experience a variety of psychological issues including fears, scanxiety, negative change in emotion and mood, doubts about their treatment and follow up, living with uncertainty, lack of understanding from others or healthcare professionals, and constantly being reminded of their illness. In addition, GIST patients may also face social issues within their social activities, relationships, work, financial situation, and, when younger, have concerns about fertility and parenting. Although this study only included locally advanced and metastatic GIST patients, part of the psychological and social challenges may also be experienced by patients on (neo-)adjuvant imatinib. The main difference between both groups is the fact that there is no perspective of being cured for advanced and metastatic GIST patients, therefore, challenges related to doubts and uncertainties may be more present and persistent in this group of patients.

Our study was the first to include the perspective of medical oncologists on the psychological and social issues that GIST patients may experience during long-term TKI treatment. Medical oncologists were well aware of some frequently patient-reported issues, such as fear of disease progression, scanxiety, and the impact on relationships. Notably, other reported issues (e.g. negative changes in emotion and mood, lack of understanding and giving up hobbies) were clearly underreported and hardly recognized by medical oncologists. This may be due to the fact that medical oncologists tend to focus more on the clinical outcomes and the physical side effects of treatment.

Some of the expressed psychological issues in our study are described in previous research among GIST patients, such as fear of progression, fear of treatment failure and uncertainty [8, 12, 19, 20]. Custers et al. [8] specifically studied fear of cancer recurrence or progression in GIST patients and found that patients with high fear of recurrence or progression experienced significantly higher levels of psychological distress, functional impairments and difficulty making plans for the future compared to patients with lower levels of fear. Macdonald et al. [20] explored the experiences and perspectives on the GIST patient journey and revealed that patients with GIST progressed through periods of crisis, hope, adaptation, new normal and uncertainty. The stage of uncertainty included fear of and/or experience of treatment failure that precipitated anxiety about the disease. Living with uncertainty and an unsettled future proved to be burdensome for GIST patients [12]. Feelings of uncertainty due to drug resistance, disease progression and the possibility of early death were experienced as challenging by half of the participants. Patient advocacy groups can play an important role in this; they can bring GIST patients into contact with each other, so they can exchange experiences and information, but also support each other. In line with our study, the participants also described lack of understanding as they pointed out that it was difficult for other people to understand how they, while not looking ill, needed to take steps to ensure they had enough energy to be sociable and active [12].

The majority of patients and medical oncologists expressed scanxiety. Scanxiety is underreported in research, but widely present in cancer clinical care. For this reason, Custers et al. [21] aimed to raise awareness for this ‘Sword of Damocles’ scan-related issue, in which recurrence or progression of disease continues to hang over patients and their families for the rest of their life. In another group of cancer patients, patients with non-small-cell lung carcinoma, scanxiety was associated with impaired quality of life. Interestingly, the severity of scan-associated distress was not associated with time since diagnosis [22], suggesting that scanxiety is a persistent issue. A qualitative study of Bui et al. [23] stated that patients accept scanxiety, which is often related to the scan result rather than the scan procedure itself. Therefore, an important next step is to define the triggers and causes for scanxiety, to enable medical oncologists to best guide and support their patients.

In our study, two thirds of the patients experienced a negative change in emotion or mood. Most patients described easily becoming emotional, which they assigned to their TKI treatment. Only a few patients felt down or depressed. Depression may be a more cancer-generic problem, affecting up to 20% of the cancer patients [24] compared to 5% of the general population [25]. Depression is often overlooked by medical oncologists in both palliative-care and non-palliative-care settings [26]. This could also be the case in GIST patients as half of the medical oncologist did not mention depression in our study. Attention for depression is relevant because when depressive symptoms are picked up, patients can benefit from treatment, which may reduce symptoms and improve their quality of life.

The study of Fauske et al. [12] was the first study to describe how living with metastatic GIST and its treatment posed challenges in relation to everyday life. In line with our study, patients emphasised how this affected multiple facets of daily life, including family life, vocational life and social life. They described how side effects as fatigue or lack of energy made many participants either avoid social activities altogether or adapt to enable them to socialise. Also, in our study, the TKI-related side effects often lead to the inability to perform certain activities, hobbies or work and forced patients to adjust their social lives.

The strength of our qualitative study is that it provides a unique and detailed insight into how GIST patients themselves experience their lives with a locally advanced or metastatic GIST while depending on life-prolonging TKI treatment. Besides side effects of treatment and sometimes consequences of previous surgery, these GIST patients also cope with psychological and social issues, which are often not addressed during their follow-up care. These issues could also have an impact on the patient’s life and can lead to a poorer HRQoL. By gaining more insight into the psychological and social issues, medical oncologists may be more aware of and recognise these issues in their GIST patients, which presents an opportunity to discuss these issues during follow-up, or if necessary, adjust their follow-up care accordingly. However, there are always limiting factors to discuss those issues with patients, of which ‘time’ is one of the most important. On the other hand, there are ways to overcome this, for example by implementing relevant psychological and social issues in a questionnaire that patients can complete before their scheduled follow-up visit [27]. With the information coming from this questionnaire the medical oncologist knows better what issues to focus on during the follow-up visit, or can provide additional information or tools to cope better with these issues, or if necessary, refer to more specialised care.

Some limitations need to be taken into account. First, the study only recruited GIST patients from the Netherlands, which may limit the generalisability of this study. To partly overcome this limitation, we interviewed medical oncologists from various countries, to explore whether GIST patients in other countries experience different psychological and social issues compared to GIST patients in the Netherlands, which did not seem to be the case. Second, only medical oncologists were interviewed. Although, all medical oncologists had ample experience delivering care to this specific group of patients, including a specialist nurse or psychologist could have provided additional information. Third, the small sample size, as compared to quantitative research. Nevertheless, we continued the recruitment of participants until data saturation was reached and have gained in-depth information in this way. Lastly, patients were long-responders to either first-line imatinib or second-line sunitinib. None of the patients were treated with further lines of TKI therapies (i.e. regorafenib, ripretinib). This is in fact an actual representation of the real world, as most long-term GIST patients are being treated with imatinib or eventually sunitinib. Next to that, we included one patient that was only 13 years of age at diagnosis, this might raise questions because young patients are more likely to have mutations that are not sensitive to imatinib. However, we did not collect data on mutational status, and as this patient responded to imatinib for 11 years, he was considered a long-responder.

## Conclusion

The impact on GIST patients’ HRQoL is determined by more than merely physical issues due to TKI-related side effects. The reported psychological and social life challenges in our study can also to some or more extent hamper the overall quality of life of GIST patients on long-term TKI treatment. To ensure optimal care for this patient group, it is essential to take the patient’s perspective into account, both in research and clinical practice. Currently, some issues are hardly recognized as they were clearly underreported by medical oncologists. By creating more awareness for these issues, and by finding ways to implement this knowledge in the current follow-up care, we can not only improve the care for this patient group, but also their quality of life.

## Supplementary information

ESM 1 (DOCX 18 kb)

ESM 2 (PDF 505 kb)

ESM 3 (DOCX 20 kb)

ESM 4 (DOCX 18.8 kb)
