# Supplementary material for: Psychological and social challenges of patients with locally advanced and metastatic gastrointestinal stromal tumours (GIST) on long-term treatment with tyrosine kinase inhibitors: a qualitative study with patients and medical oncologists
Source: Support Care Cancer. 2023 May 26;31(6):352. doi: 10.1007/s00520-023-07810-7 (PMC10220127; doi:10.1007/s00520-023-07810-7)
Supplement: Supplementary file 1 — (DOCX 18 kb) [file 520_2023_7810_MOESM1_ESM.docx]

**Supplementary material 1 – The patient and medical oncologist semi-structured interview guides**

**Patient Interview Guide**

**Outline**

- Introduce the study and the reasons we are asking patients to participate
- Assure the participant about maintaining confidentiality
- Explain the need to gather some personal information about them for the research study
- Complete the first part of the CRF
- Ask them about (current) issues/ experiences they are having as a consequence of the GIST diagnosis and TKI treatment
- Complete the last part of the CRF
- Thank them for sharing their experiences with us

**GENERATION OF RELEVANT HEALTH-RELATED QUALITY OF LIFE ISSUES**

***(Italic font indicated interviewer’s script)***

- **Introduce the study and the reasons we are asking them to participate**

*We are asking for your help in researching the experiences of people who have been diagnosed with GIST. We have a special interest in patients with GIST who are on prolonged treatment with drugs such as imatinib, sunitinib or regorafenib. I would like to ask you a few things about your health.*

- **Assure them about maintaining confidentiality**

*All of the information you are providing will remain confidential and your data will be assigned a unique research number so that your name will not appear anywhere.*

- **Explain the need to gather some personal information about them for the research study**

*In order to provide some background information for our research I would like to gather some personal information about your life circumstances including current work and relationship status, and about your health (e.g. date of diagnosis; past and current treatment; time since last treatment).*

- **Complete the first part of the CRF**

*The CRF is a form used to collect further information about your disease and your treatment.*

- **Ask them about their illness and about their experiences in the four following periods** (use follow-up questions if the answer is incomplete or indefinite)

*Can you tell me about your illness/condition?*

*What are the most important things you experienced?*

- *From the time you first got symptoms*
- *When you got the diagnosis*
- *About the treatment initiation and your experiences while on treatment*
- *Until your current situation*

It may be useful to prompt the patient to consider specific domains of the biopsychosocial model.

Some examples are shown:

- *What are you not able to do that you would formerly do before your illness, any why?*
- *What symptoms do you experience as a consequence of GIST or its treatment?*
- *Are you limited in normal daily activities (e.g. work, household, shopping, taking care of the children, sports) compared to before your illness? What is it that limits you?*
- *Are you undertaking fewer social activities (e.g. hobbies, meeting up with friends) and why?*
- *Have changes in relationships with family/friends occurred?*
- *Do you have financial problems or worries due to your illness (e.g. insurance, mortgage, loans)?*
- *Have your personal feelings changed (e.g. satisfaction with life, spirituality)?*
- *Has your emotional wellbeing changed (e.g. feelings of anxiety or worrying)?*
- *Are there any other issues or comments you would like to make regarding your illness and treatment and your quality of life?*

The following questions can be used to explore the experience of living with advanced or metastatic GIST, its unknown prognosis and chronic treatment for GIST.

- *Could you tell me about your relationship regarding the medication (“the pill”)?*
- *Could you tell me about your side effects? How do they impact your life?*
- *Do you consider stopping with your medication for GIST? Why yes/no?*
- *If not what would be requirements to consider stopping your medication in your opinion?*
- *After what period of treatment you might consider stopping your medication for GIST?*
- *How do you experience living with chronic cancer (GIST)? Has is changed over time and how?*
- *How has living with chronic cancer (GIST) affected you as a person?*
- *How do you see your future with chronic cancer (GIST)?*
- *Do you consider yourself to be healthy or ill? And why?*
- **Complete the last part of the CRF**

*Complete the last questions of the CRF regarding the participant’s opinion about stopping or continuing their medication.*

- **Thank them for sharing their experiences with us and completing the CRF**

*Thank you very much for your time today and for agreeing to participate in this research study. Your personal information and experiences are very valuable to us in the research of quality of life in patients with GIST.*

**Medical Oncologist Interview Guide**

**Outline:**

- Introduce the study and the reasons we are asking medical oncologists to participate
- Ask the medical oncologist about issues/experiences metastatic GIST patients are facing because of their GIST diagnosis and TKI treatment.
- Thank them for sharing their experiences with us.
- **Introduce the study and the reasons we are asking them to participate**

*First of all, I am very grateful that you are willing to participate in this interview. Before we start, I will explain more about the interview and the focus of the interview. My research project is about long-term survivorship challenges of advanced/metastatic GIST patients who are treated with Imatinib or other TKIs for 5 years or longer. These long-term responders are the GIST patients we want to focus on during this interview. In this interview, I would like to ask you about what issues these patients experience after their diagnosis and during their treatment.*

- **Ask the medical oncologist about issues/ experiences metastatic GIST patients are facing because of their GIST diagnosis and TKI treatment.**

*How many advanced/metastatic GIST patients you see on a yearly basis in your outpatient clinic?*

- *How many are long-term responders?*
- *Are most patients treated with Imatinib or are there also patients responding long time to other TKIs?*

*What physical complains/symptoms/side effects do patients experience during their FU?*

- *Do symptoms change over time? Do you see other side effects at the start of treatment than after 5-years of treatment?*
- *Do side effects differ between different TKIs?*
- *Are patients limited in their normal daily activities (e.g. work, household, shopping, taking care of the children, sports)?*
- *Are there patients who can no longer do what they could compared to before their GIST diagnosis?*

*Do patients experience psychological problems as a consequence of the diagnosis or treatment?*

- *Is there an emotional impact?*
- *Do patient worry and discuss their worries during FU? What do they worry about?*

*Are patients undertaking fewer social activities (e.g.-hobbies, meeting up with friends) and why?*

*Do you see patients were relationships with family/friends have changed completely as consequence of diagnosis/treatment?*

*Do patients have financial problems or concerns due to their treatment?*

*How do you think the quality of life is in general in these patients?*

*Do you think patients consider themselves healthy or ill?*

*Do you think patients always take their medication?*

- *A specific reason not to take their TKI? Mostly due to toxicity?*

*Do patients consider cessation of their TKI treatment?*

- *In certain situations or after a certain time?*
- *After stopping, do you see rapid progression or do patients remain stable?*
- **Thank them for sharing their experiences with us.**

*Thank you very much for your time today and for agreeing to participate. Your information is very valuable.*
