# Supplementary material for: Psychological and social challenges of patients with locally advanced and metastatic gastrointestinal stromal tumours (GIST) on long-term treatment with tyrosine kinase inhibitors: a qualitative study with patients and medical oncologists
Source: Support Care Cancer. 2023 May 26;31(6):352. doi: 10.1007/s00520-023-07810-7 (PMC10220127; doi:10.1007/s00520-023-07810-7)
Supplement: Supplementary file 3 — (DOCX 20 kb) [file 520_2023_7810_MOESM3_ESM.docx]

**Supplementary material 3 – Overview of the psychological issues and subthemes expressed by the participants**

| Psychological issues | Patients (n=15) | Medical oncologists  (n=10) | Total  (n=25) |
| --- | --- | --- | --- |
| Fears | **12** | **8** | **20** |
| - Fear of long-term side effects and regular CT scans | 5 | - | 5 |
| - Fear of death | 5 | 1 | 6 |
| - Fear of disease progression or recurrence | 4 | 6 | 10 |
| - Fear of disease activity when experiencing a physical sensation | 3 | 2 | 5 |
| - Fear of medication getting too expensive | 2 | - | 2 |
| - Fear of resistance or not responding to treatment | 8 | 3 | 11 |
| Scanxiety - Feeling anxious around tests, scans and follow-up visits | **10** | **9** | **19** |
| Constantly reminded | **4** | **4** | **8** |
| - Due to having to take medication (daily) | 1 | 2 | 3 |
| - Due to comments from others | 1 | 2 | 3 |
| - Due to (daily) side effects of treatment | 2 | 1 | 3 |
| - Due to the regular scans and follow-up visits | 1 | 1 | 2 |
| Doubts | **7** | **4** | **11** |
| - Doubts about the frequency and type of follow-up scans | 2 | - | 2 |
| - Doubts about treatment due to the experienced side effects | 3 | - | 3 |
| - Doubts about continuing treatment | 4 | - | 4 |
| - Doubts about stopping treatment | 5 | 4 | 9 |
| Negative change in emotion and mood | **10** | **8** | **18** |
| - It is stressful to live with an incurable (chronic) cancer | - | 2 | 2 |
| - Feeling down | 1 | 5 | 6 |
| - Feeling depressed | 3 | 4 | 7 |
| - Mood swings | 2 | - | 2 |
| - Feeling irritated (because I have to repeat my story) | 1 | - | 1 |
| - Feeling frustrated (because of the side effects) | 3 | - | 3 |
| - Easily becoming emotional | 5 | - | 5 |
| - Loss of temper | 3 | - | 3 |
| - Having more emotional off-days | 2 | - | 2 |
| Lack of understanding | **7** | **2** | **9** |
| - By health care professionals | 4 | - | 4 |
| - By others | 4 | 2 | 6 |
| Living with uncertainty | **7** | **2** | **9** |
| - No carefree life | 2 | 1 | 3 |
| - Uncertainty about the future | 3 | 1 | 4 |
| - Uncertainty about the course of my disease | 5 | 2 | 7 |
