# Supplementary material for: Psychological and social challenges of patients with locally advanced and metastatic gastrointestinal stromal tumours (GIST) on long-term treatment with tyrosine kinase inhibitors: a qualitative study with patients and medical oncologists
Source: Support Care Cancer. 2023 May 26;31(6):352. doi: 10.1007/s00520-023-07810-7 (PMC10220127; doi:10.1007/s00520-023-07810-7)
Supplement: Supplementary file 4 — (DOCX 18.8 kb) [file 520_2023_7810_MOESM4_ESM.docx]

**Supplementary material 4 – Overview of the social issues and subthemes expressed by the participants**

| Social issues | Patients (n=15) | Medical oncologists  (n=10) | Total  (n=25) |
| --- | --- | --- | --- |
| Impact on social activities | **10** | **9** | **19** |
| - Had to negatively adjust social activities | 3 | 2 | 5 |
| - Had to change or give up my hobbies | 5 | 1 | 6 |
| - Difficulty going out in public because of having to deal with my disease or the side effects | 3 | 7 | 10 |
| - Always having to plan activities and/or planned activities are always subject to change | 3 | - | 3 |
| - Unable to function in big groups | 2 | - | 2 |
| Relationships | **10** | **9** | **19** |
| - Lost friends | 3 | - | 3 |
| - Relationship difficulties with your partner | 2 | 8 | 10 |
| - Not able to find a partner | 2 | - | 2 |
| - Feeling isolated | 2 | - | 2 |
| - Feeling a burden to others | 6 | 2 | 8 |
| Fertility and parenting | **3** | **1** | **4** |
| - Worried about becoming infertile and not being able to have children | 1 | 1 | 2 |
| - Difficulty starting a family | 2 | - | 2 |
| - Worried about not being able to be a role model for my children | 1 | - | 1 |
| Financial difficulties | **6** | **3** | **9** |
| - Less income | 2 | 2 | 4 |
| - Worried about not being able to pay for everything | 3 | 1 | 4 |
| - Not able to buy a house | 2 | - | 2 |
| - More expenses on health insurance | 3 | - | 3 |
| Work | **6** | **4** | **10** |
| - Not able to work full-time | 3 | 3 | 6 |
| - Change job | 3 | - | 3 |
| - Lost my job | 1 | 3 | 4 |
| - Feeling as if you have no career opportunities | 2 | - | 2 |
